# The Amalgam Question

**Published:** 1861-09

**Authors:** 


DENTAL REGISTER OF THE WEST.

VOL. XV.] SEPTEMBER, 1861. [No. 9.

### Original Essays and Communications.

### THE AMALGAM QUESTION.

THAT various morbid symptoms may be produced by what is commonly called mercurial poisoning is no longer doubted; but it is still a question whether or not free mercury is poisonous. Christison and others maintain that it is not; while Orfila, Buchner, Pereira, and others maintain the opposite. The question is, however, of but little practical moment; for all these, and everybody else, agree that many, if not all, of the compounds of mercury are poisonous. It is well known that the *metal* applied externally, or inhaled in the form of vapor, will produce constitutional results; but whether or not it loses its metallic state, by combination with some other element, before it acts thus on the system, is not yet determined.

Salivation, ulceration and sloughing, and shaking palsy (*tremor mercurialis*) are the diseases most frequently produced by the action of mercury; and of these, the first is much the most frequent. The last mentioned is usually produced by the long continued and very gradual introduction of the drug into the system. Gilders, barometer and looking glass manufacturers, and workers in quicksilver mines are frequently affected with this disease; and, if their occupations are not abandoned, more formidable, and usually fatal diseases supervene.

An important practical question arises, as to how much of the metal, or how much of any one of its compounds, is necessary to produce observable constitutional effects. And here it is well known that striking differences will be manifested by different constitutions. Some persons can take large mercurial doses without inconvenience, while others are poisoned by very minute quantities. Accidental causes, no doubt, have often great influence; and the whole question is, to some extent, involved in difficulty, as we are not able to define, positively, the mode of action of this powerful drug. It is evident that the metal is absorbed, and deposited in some part of the system, or is thrown out by excretion. And as there may be great variations in the absorbing and excreting powers of the same person, at different times, it is not strange that the same constitution is more susceptible of the action of mercury some times than others. Mercury, according to Schubarth, Weller, and others (see Christison on Poisons), has been detected in the blood, in the sweat, the saliva, in the urine, the bile, the intestinal secretion, and in the fluid of ulcers. It has also been found, in its metallic state, in the bones, brain, pleura, lungs, cellular tissue, synovial tissues, the humors of the eye, etc. And it is evident that its constitutional effects are owing to its absorption; for these are the same, whether the mercurial is applied to the skin, to the mucous membrane, or injected into the veins.

Bearing these things in mind, (and they are the views of the friends of mercury,—not those of its enemies) it is not unreasonable that we should, at least, be cautious in the use of an agent so powerful. And with this view, it may again be asked, how small a quantity of any mercurial is capable of producing observable constitutional effects.

In Pereira's Materia Medica, vol. 1, page 594, note, a case is described, in which there was, evidently, a strong idiosyncrasy against the use of mercury. "A patient of Mr. G.'s, of the Borough, desired him never to give her any mercury, as that drug was a poison to her whole family, to which he, without arguing the point, at once assented. In Mr. G.'s absence, the late Mr. C. was consulted as to some trifling disorder of the bowels, and, not knowing the peculiarity of his patient's constitution, prescribed two grains of calomel. The next morning the lady showed the prescription to Mr. C., saying that she was sure she had taken mercury, as she felt it in her mouth. In a few hours ptyalism ensued; in consequence of which she lost her teeth, her jaw exfoliated, and she ultimately, after a succession of ailments, died, in about two years." According to Culerier, six or eight grains of mercurial ointment have often sufficed to excite violent salivation. Usually, half the weight of this ointment is mercury. Of the black oxyd of mercury, the dose is from half a grain, to two or three grains; and salivation is often readily produced by the administration of a few doses.

When calomel is administered as an alterative, the usual dose is from half a grain to a grain, and it is well known that observable constitutional effects often result from these doses. Pereira refers to the case of a lad, aged 14, "in whom six grains of calomel apparently produced inflammation and ulceration of the mouth, enormous swelling of the face, mercurial fetor of the breath, mortification, and death. There was no ptyalism." This is an *exact* description of a case that occurred in my own practice, in 1845, except that the patient was a *girl*, *aged* 12.

The dose of chloride of mercury (corrosive sublimate) is from one-sixteenth to one-eighth of a grain; and, if administered in quantities much greater than these, it is apt to gripe and purge.

From these observations and quotations, it would be readily inferred that, at least, some of the mercurial compounds are very active and powerful medicines or poisons, as the case may be. Indeed, we have seen many cases of severe salivation, caused, respectively, by a single blue pill of three grains I know that such cases are regarded as exceptional; but they are so frequent that their claim to such regard is scarcely valid.

It is well known that mercury is but slightly vaporized at ordinary temperatures. Not only is the quantity of vapor formed small in quantity, in proportion to the surface exposed, but, being nearly seven times as heavy as air, it rises but a small distance from the surface. If gold leaf is suspended over mercury, it will be whitened by amalgamation to the height that the vapor rises. The barometer maker's danger is, therefore, mainly from contact with the skin, and not from inhalation. As this contact is not extensive, the mercury must be very slowly introduced into the system. Yet it is well known that his occupation is not a safe one. That a very slow and greatly prolonged introduction of this metal into the system is capable of producing injurious results, is a well recognized fact among scientific men. Dr. Scheele reports " a fatal case, attended with salivation, brought on from wearing at the breast, during six years, a leathern bag, containing a few drams of liquid mercury."

But this is the "amalgam," not the *mercurial* question; and we, therefore, proceed to inquire, in view of all the facts known to science, whether or not it is at all reasonable that mercurialization should result from amalgam fillings in the teeth.

Amalgam plugs are usually large, as none but quacks insert them in small cavities. We have weighed some freshly inserted, and many that were considerably corroded, and have found a number weighing over forty grains each. Many are much smaller. We have many times seen two, three, or four large ones in the same mouth; and in one mouth we saw *seventeen*, large and small. But, for illustration, let us suppose a case in which eighty grains of amalgam cement are inserted. This is not an extraordinary case. Four molars, with a small plug each, would fill the bill. If the silver amalgam is used, about one-half the weight is mercury. If the silver and tin, the proportion of mercury is not quite so great. For convenience of calculation, let us take the former.

The forty grains of mercury (even though inert in the metallic state, which is not yet proved, however,) would make two drams of blue mass, or forty officinal blue pills. They would make about fifty-four grains of corrosive sublimate. They would yield forty-seven grains of calomel, or nearly forty-two grains of black oxyd of mercury.

Now no scientific man could be surprised at witnessing constitutional effects from the presence of such quantities of any one of these drugs.

But the amalgam advocates may, and do object that these compounds are not liable to be formed in the mouth; but, with the next breath, they go on to lament the "blackness," "discoloration," "coloration," etc., through all the changes, ascribing it all the while to *oxydation*, thus acknowledging that the last named drug is almost invariably formed. And it is objected, too, that if formed at all, these drugs are formed, and, therefore, introduced so slowly and gradually, that they can produce no perceptible effects. But such objectors manifest an ignorance of scientific truth hardly excusable in this enlightened age of the profession. The slow and gradual introduction is the important point to be considered. It is here that the danger lies. When rapidly introduced, the system is aroused, and rebels; and much of the poison is ejected. This slow introduction is nothing less than "nurturing up wrath against the day of wrath," as in the case of the man that wore the metal in a leathern bag. The poison could only pass infinitesimally into his system; yet in six years it did its work. And those who wear amalgam plugs in their mouths for six years, and especially for "fifteen years," have no security that their fates will not be similar. Indeed, many cases occur in which there is severe mercurial disease, while neither physician nor patient suspects the cause. The physician is deceived by the patient's assertion that he has taken no mercurials, or perhaps no medicines of any kind. Many cases that pass for "spontaneous salivation" are the legitimate result of the presence of amalgam plugs in the mouth. A case of "shaking palsy" occurred in our practice in 1850, which resisted treatment for two months, growing worse all the time, and which recovered promptly, without farther treatment, after removing several amalgam plugs from the teeth. And we must be excused if, when we read of old practitioners, whose neighbors, as well as themselves, have been, all along, using amalgams, and who yet assert that they have never seen a case of ptyalism, or other constitutional disease, arising from their use—we must be excused, if we look upon them "with considerable doubt as to the value of their judgment or opinions, as reliable diagnosticians."

It would be amusing, were the case not serious, to witness the varied positions, and the arguments to sustain these positions, resorted to by those who deny the fact of mercurial poisoning by amalgam plugs.

One believes "the profession had never heard of but *one* practitioner who *thought* that the result (ptyalism) was produced by amalgam." But this only proves that he has not listened through the ears of the profession, perhaps not even with professional ears.

Another can not believe that amalgam fillings can produce ptyalism, because this is produced through the general system, whether the mercury is used externally or internally. Now it is not probable that any one believes that amalgam plugs can produce ptyalism by mere local action.

Another is a disbeliever, because "it is well known that mercury, uncombined, is inert"—which is merely an assertion, and because "equally so must it be when combined with silver or tin,"—which is a mere assumption. And he is farther confirmed in his position, from the fact that "the protochloride of mercury (calomel) and deuto-chloride of mercury (corrosive sublimate) are formed from sulphate of mercury and muriate of soda, triturated and sublimated. As this process can not very well be carried on in the mouth, it is hardly supposable that they are elaborated to any extent." Well, there is chemistry for you ; but it is

> " Science distorted and torn into bits,
> And tortured and frightened half out of her wits."

Are we to infer that chlorine and mercury can combine only under the circumstances here detailed ?

It is rather surprising, and quite mortifying to witness the lack of knowledge manifested by many of the profession, in regard to the obvious and ordinary constitutional effects of mercury. When it is objected to the use of amalgams that there is danger of mercurial poisoning, the answer is that abscess, exostosis, and necrosis occur in mouths where no mercury is used, as if these were what is meant by *constitutional* effects of mercury. And where a genuine case of poisoning is presented, it is referred to some other cause than mercury, because "many cases of irritated gums looking terribly enough had yielded to proper constitutional and local treatment," as if irritated gums were all of ptyalism, and as if acute ptyalism were not amenable to treatment. Any scientific dentist would infer that there is greater danger of mercurialization, from this source, when the fluids of the mouth are acid, than when they are alkaline. If a case of ptyalism presented itself, and the amalgam plugs were allowed to remain, a part of the proper treatment would be to secure an alkaline state of the saliva. And he would infer that the disease was, most likely, the result of either the oxydation or chloridation of mercury ; and as its compounds with sulphur are far less poisonous than its oxyds or chlorides, and are nearly insoluble, he would take such measures as would secure its sulphidation. We make these remarks, merely to remind the reader that even ptyalism is amenable to proper constitutional treatment ; and hence, yielding to treatment is no evidence that the disease is not ptyalism. If the corrosion of the mercury is stopped for the time, the disease will usually exhaust itself, and recovery will take place without direct treatment.

One disbeliever suggests that many of the cases taken for the effects of mercury are the result of "mechanical irritation,—which would have resulted just as soon from an equally bad gold plug." Now every one who understands the subject knows that *mechanical* irritation never did, and never will produce results very much like mercurial ptyalism.

It is well known that ptyalism may be, and is produced by other causes than mercury. There is what is called "spontaneous ptyalism." And it is cordially admitted that in a large majority of cases, in which amalgams are used, no observable constitutional effects result. But it will not do, on this account, to deny the fact of mercurial poisoning by amalgam plugs. The same warrant is afforded for the denial of mercurialization from any source. In a large majority of cases in which mercurials are administered, no poisoning is observable. Indeed, it would be no more than consistent for some of our disbelieving brethren to write an article to prove that both mercury and its compounds are inert. They would be at no loss for arguments stronger than those they are in the habit of using in discussing the "amalgam question." Why, a patient has taken over fifty drams of calomel, in less than as many hours, "without the least sensible effect." Take the position, aud *stick to it*, that calomel never produces ptyalism. And if it should occur while the patient is taking the drug, be firm and *consistent*, by claiming that it was about to occur any way, and has resulted simply from "mechanical irritation." W.

